# Human cellular mitochondrial remodelling is governed by miR-2909 RNomics

**DOI:** 10.1371/journal.pone.0203614

**Published:** 2018-09-25

**Authors:** Deepti Malik, Deepak Kaul

**Affiliations:** Molecular Biology Unit, Experimental Medicine and Biotechnology, Postgraduate Institute of Medical Education and Research, Chandigarh (India); University of South Alabama Mitchell Cancer Institute, UNITED STATES

## Abstract

**Background:**

There exists a general recognition of the fact that mitochondrial remodelling as a result of aerobic glycolysis ensures human somatic cells to revert to a more primitive-form exhibiting stem-like phenotype. The present study is an attempt to demonstrate that miR-2909 RNomics within human peripheral blood mononuclear cells (PBMCs) has the inherent capacity to re-program these cells to exhibit mitochondrial remodelling paralleled by aerobic glycolysis together with intracellular lipid inclusions. Such re-programmed PBMCs also expressed genes having ability to sustain their de-differentiation state and survival.

**Material and methods:**

Human PBMCs were programed to ectopically express miR-2909. Expression levels of genes including glucose transporter-1 (*Glut-1)*, hexokinase (*HK)*, hypoxia inducia factor-1 *(HIF-1α)*, *c-Myc*, *p53*,mechanistic target of rapamycin complex *(mTORC1*), polycombcomplex protein (*Bmi-1)*, *Notch*,*Nanog*,*Tie-2*, *Oct-4*,*CD59*, *p53*, *CD34*, B-cell lymphoma-2 (*Bcl2)*,sterol regulatory element-binding protein2 (*SREBP2*), peroxisome proliferator-activated receptor gamma *(PPARγ*) nuclear respiratory factor 1 (*NRF1*), mitochondrial transcription factor A (*Tfam*), peroxisome proliferator-activated receptor gamma coactivator 1-alpha (*PGC1α*) within miR-2909 expression vector transfected human PBMCs as well as PBMCs transfected with control vector containing scrambled sequence after 48h post-transfection using RT-qPCR and cellular ultrastructural features induced by miR-2909 ectopic expression were observed using transmission electron microscopy and morphometric analysis of an electron micrograph.

**Results:**

Ectopic expression of miR-2909 within human PBMCs resulted in their reprogramming into stem-like phenotype indicated by mitochondrial globular shaped coupled with cristae-poor morphology. Nuclear to cytoplasmic ratio (N/C), quantification of ATP levels, GSSG/GSH ratio, mitochondrial cytochrome *c* oxidase activity, secreted lactate concentrations, activity of antioxidant enzymes, levels of esterified cholesterol and triglycerides and flow-cytometric detection of apoptosis confirmed the compromised nature of mitochondrial function induced by ectopic miR-2909 expression in human PBMCs.

**Conclusion:**

Based upon these results we propose that AATF gene-encoded miR-2909 may act as an epigenetic switch for cellular aerobic-glycolysis to ensure de-differentiation.

## Introduction

Although the underlying epigenetic mechanism that governs the reprogramming of somatic cells into a stem-like phenotype is far from clear several recent findings have revealed that mitochondrial maturation and remodelling drives these somatic cells into either a differentiated or dedifferentiated state [1]. Upon reprogramming, the differentiated cells exhibit globular mitochondrial shape and functionally immature state [1]. This phenomenon is paralleled by increased cellular expression of genes coding for mitochondrial uncoupling protein (UCP-2) which in turn ensures aerobic glycolysis by shunting pyruvate out of mitochondria leading to increased production of lactate coupled with reduced ATP level [1–5]. Interestingly, the mechanistic target of rapamycin complex (mTORC1) has been shown to ensure cellular aerobic glycolysis by inducing not only the genes coding for UCP-2, hypoxia inducible factor-1α (*HIF-1α*) and c-Myc but also promotes lipogenesis through the upregulation of sterol regulatory element-binding protein2 (SREBP-2) gene [6,7]. The tumor suppressor p53 has attracted resurgence of interest because of its ability to inhibit mTORC1 [8] as well as for its role as a potent somatic cell reprogramming barrier for the emergence of stem-like state [9,10]. A new dimension was added to the energy metabolism by the finding that revealed the ability of human apoptosis antagonizing transcription factor (AATF) gene to code for regulatory non-coding microRNA designated as miR-2909 apart from its AATF protein coding transcript [11]. Various findings revealed that mutual cooperative regulation of AATF protein and miR-2909 is responsible for governing of various cellular processes especially cell cycle progression, checkpoint control and cellular autophagy/apoptosis [12,13]. However, the complexity of this gene does not end here since AATF protein has been shown to regulate the genes coding for p53, mTORC1 and mTORC2 [13,14]. Further miR-2909-dependent upregulation of cellular UCP2 expression ensures aerobic glycolysis by decoupling of glycolysis from the aerobic respiration [3]. Keeping in view the role of mitochondrial remodelling in cellular de-differentiation, the present study was addressed to understand whether or not ectopic miR-2909 expression within human PBMCs has the ability to initiate mitochondrial remodelling, aerobic glycolysis and intracellular lipid synthesis coupled with storage in such cells thereby exhibiting expression of genes that are characteristic of stem-like de-differentiation.

## Materials and methods

### Human PBMCs: Isolation and *In-vitro* maintenance

The present study included 30 healthy volunteers who had come to donate blood in Blood Transfusion Department of our Institute hospital. Approximately 10 ml of blood was withdrawn from the subjects after their informed consent on prescribed format approved by our Institutional Ethics Committee, Postgraduate Institute of Medical Education and Research (PGIMER), Chandigarh. The Institutional Ethics Committee evaluated and provided ethics clearance/approval for the submitted project and the design of this study in accordance with the principles outlined in the declaration of Helsinki [15]. The inclusion criteria for the selection of healthy subjects were age ranging from 25–40 years with body mass index (BMI) range 18.5–25 kg/m2. Drug abuse and subjects suffering from any chronic disease were excluded in the study. PBMCs were isolated by Ficoll density-gradient centrifugation from 10 ml blood collected in vacutainer EDTA tubes [16]. Briefly, 10 ml of heparinized blood was layered onto 7 ml of Histopaque and centrifuged at 400xg for 40 min in swinging bucket rotors at room temperature. After centrifugation, the layer of PBMCs was aspirated and washed with sterile PBS by centrifugation at 100xg. These cells were subsequently seeded at the initial density of 2 x 10^5^ cells per well in 6 well culture plate and were maintained in RPMI-1640 culture medium containing 10% FCS, at 37°C in 5% CO_2_ atmosphere.

### Human PBMCs programed to ectopically express miR-2909

Genomic DNA was extracted from the mononuclear cells by standard phenol-chloroform procedure [17]. For construction of miR-2909 expression plasmid, precursor sequence of miR-2909 was amplified from genomic DNA using taq polymerase and appropriate primer pairs. The amplified fragment was then agarose gel purified and subsequently cloned into the pEF6/V5-His-TOPO expression cloning vector under control of EF1α promoter vector (Invitrogen, Carlsbad, CA, USA). Plasmid DNA was isolated from individual colonies. The positive clones containing miR-2909 expression construct; designated as pEF6-miR-2909 expression construct were confirmed by PCR amplification and the orientation of insert in the plasmid was verified by sequencing. For overexpression of miR-2909, PBMCs were transfected with pEF6-miR-2909 expression construct using metafectene transfection reagent (Biontex). Control PBMCs were transfected with null vector containing scrambled sequence using the same transfection reagent. Assessment of transfection efficiency was determined after harvesting the cells after 48h by comparing the expression of miR-2909 in cells transfected with pEF6-miR-2909 expression construct with control using qRT-PCR. Non-coding small nuclear RNA U6 was used as an invariant control for exploring the expression of miR-2909 within human PBMCs.

### Cellular miR-2909 and its effector gene expression

Keeping in view our recent findings [3] regarding the inherent capacity of miR-2909 RNomics to decouple glycolysis from aerobic respiration through the upregulation of mitochondrial coupling protein (*UCP2*), the present study was directed to explore whether or not the miR-2909 RNomics has the ability to regulate genes that play crucial role in cellular energy metabolism (*Glut-1*, *HK*, *HIF-1α*, *c-Myc*, *p53*, *mTORC1*), dedifferentiation (*Bmi-1*, *Notch*, *CD59*, *p53*, *Nanog*, *Oct-4*, *Tie-2*), apoptosis (*Bcl2*) and intracellular lipid synthesis and storage (*SREBP2*, *PPARγ*, *mTORC1*), transcriptional regulators of mitochondrial OXPHOS genes (Tfam, NRF1, PGC1α).The primers’ sequences used in this study are listed in S1 Table. Total RNA including the small RNA was extracted from miR-2909 expression vector transfected human PBMCs as well as PBMCs transfected with control vector containing scrambled sequence after 48h post-transfection using miRNeasy mini kit in accordance with the manufacturer’s instructions. cDNA synthesis was performed via miScript Reverse transcription kit as per suppliers’s instructions. Gene expression was assayed using miScript SYBR Green Mix and the Real time PCR (Stratagene, San Diego, CA, USA). GAPDH were used as an invariant control for normalizing the expression of various genes. The 2-ΔΔCT method was used to calculate the relative expression of target genes. Total cellular protein was extracted using Laemmli’s buffer [18] and the protein levels of dedifferentiated marker CD34 and β-actin were determined through western blotting using appropriate antibodies. β-actin antibody was used as an invariant control. Scion Image Analysis software was used for densitometry analysis and the results were expressed as intensity ratio of target protein to β-actin protein taken as arbitrary unit.

### Influence of miR-2909 on mitochondrial function

To scrutinize the role of miR-2909 on mitochondrial function, various parameters include quantification of ATP levels, mitochondrial cytochrome *c* oxidase activity and secreted lactate concentrations in human PBMCs transfected with either miR-2909 expression vector or control vector after 48h post-transfection using their respective kits. Cells were lysed using cell lysis buffer supplied by Biovision. ATP levels were quantified using ATP kit (ATP colorimetric/fluorometric assay kit, BioVision) according to the manufacturer's instructions. ATP values were normalized to protein concentrations quantified with the Bradford assay protocol [19]. Mitochondrial cytochrome *c* oxidase activity was measured using the cytochrome *c* oxidase assay kit supplied by Biovision using automatic plate reader (Infinite M200 Microplate Reader; Tecan).Bradford assay was used to quantify the protein concentration. Secreted lactate concentrations in cell culture media of control and miR-2909 transfected PBMCs were determined by using L-Lactate Assay Kit (Cayman Chemical, Ann Arbor, Michigan, USA). The fluorescent product was analyzed with an excitation wavelength of 530–540 nm and an emission wavelength of 585–595 nm using automatic plate reader (Infinite M200 Microplate Reader; Tecan). Lactate values were normalized to protein concentrations quantified with the Bradford assay protocol [19].

### Influence of miR-2909 on cellular oxidative stress and apoptosis

In order to study the influence of cellular miR-2909 upon oxidative stress, the ratio of glutathione oxidized (GSSG) to glutathione reduced (GSH), activity of antioxidant enzymes; superoxide dismutase (SOD), reactive oxygen species (ROS) coupled withnitric oxide (NO) production were measured in human PBMCs that were ectopically overexpressing miR-2909 as well as human PBMCs transfected with null vector containing scrambled sequence. GSSG to GSH ratio was determined using GSH/GSSG Ratio Detection Assay Kit II; Fluorometric—Green (Abcam). GSH (reduced) and total GSH (GSH + GSSG) was measured directly with their standards provided by the kit. GSSG was then calculated by subtracting the GSH amount from the total GSH + GSSG amount, thus providing the final GSH:GSSG ratio. Fluorescence was monitored at Ex/Em = 490/520 nm with Infinite M200 Microplate Reader (Tecan). ROS was detected using the dye DCFH-DA according to the procedure described by Wu and Yotnda, 2011 [20]. Superoxide dismutase in control and miR-2909 transfected PBMCs was estimated according to the methodology proposed by Kono [21]. Inhibition in the rate of NBT reduction was measured at 540nm using Infinite M200 Microplate Reader (Tecan). BCA assay was used to determine the total amount of protein in the cell lysate. Nitric oxide production during cell culture was measured as accumulated supernatant nitrite by the Griess reagent (1% sulphanilamide diluted in 5% phosphoric acid and 0.1% *N*-(1-naphthyl ethylenediaminedihydrochloride in sterile water [20]. Absorbance at 550 nm was read on an automatic plate reader (Infinite M200 Microplate Reader; Tecan). The values of nitrite concentration in the culture samples were obtained from the standard curve. Results were expressed as nmol per mg of protein. For apoptosis assays, cells were stained with FITC Annexin V coupled with propidium iodide and apoptosis was measured using BD FACS Diva Software (BD,FACS Canto II).

### miR-2909 induced intracellular neutral lipid level

To assess the role of miR-2909 in the formation of intracellular lipid droplets, levels of esterified cholesterol and triglycerides were estimated. Control and miR-2909 transfected PBMCs were lysed using cell lysis buffer supplied by Biovision. The levels of cholesteryl esters were determined by subtracting the value of free cholesterol from the total (cholesterol plus cholesteryl esters) by using Total Cholesterol Assay Kit (Cell Biolabs). The levels of triglyceride in control and miR-2909 transfected PBMCs were estimated using the commercial kit provided by Kee Diagnostics. Total protein was estimation by BCA assay.

### Cellular ultrastructural features induced by miR-2909 ectopic expression

Cellular ultrastructural features were observed using transmission electron microscopy. After 48 h post-transfection, human PBMCs transfected with either miR-2909 expression vector or control vector were fixed in 3% glutaraldehyde in sorenson’s phosphate buffer, pH 7.2 for 4–6 hr followed by secondary fixation in 1% osmium tetraoxide in Millonig’s phosphate buffer pH 7.2. Cells were processed and embedded in Taab-812 embedding medium. Ultrathin sections of 60nm thickness were cut on Leica EMUC-6 ultra-microtome (Leica Microsystem GmbH, Wien, Austria) and taken on nickel grids. Ultrathin sections were stained with uranyl acetate and lead citrate and examined at 80kV accelerating voltage at JEOL Transmission Electron Microscope; JEM-1400 Plus (JEOL, Tokyo, Japan) equipped with XR81M-B Camera (Advanced Microscopy Techniques Corp, Woburn, MA, United States). Digital electron micrographs of control and miR-2909 transfected PBMCs were acquired using AMT Image Capture Engine V602 software (Advanced Microscopy Techniques Corp, Woburn, MA, United States. we estimated the cellular nuclear to cytoplasmic ratio and mean mitochondrial volume density (taken randomly 12 cells in both control and miR-2909 transfected cells) using Image J software equipped with XR81M-B Camera. The volume density (Vv) of mitochondria was expressed as percent volume: Vv = (Pm/Pc)*100 (%), where Pm is the pixel size occupied by each mitochondrial structure and Pc is the total cell mass in pixel size occupied by the cell [22,23].

### Statistical analysis

Statistical analyses were performed by SPSS window version 19. Data was expressed as mean ± SD of all the experiments done independently. Unpaired Student’s T test was used for comparison between two groups. The difference was considered statistically significant when *p* < 0.01 and p<0.05.

## Results

### Somatic cellular ectopic miR-2909 expression and effector genes

**Fig 1:**
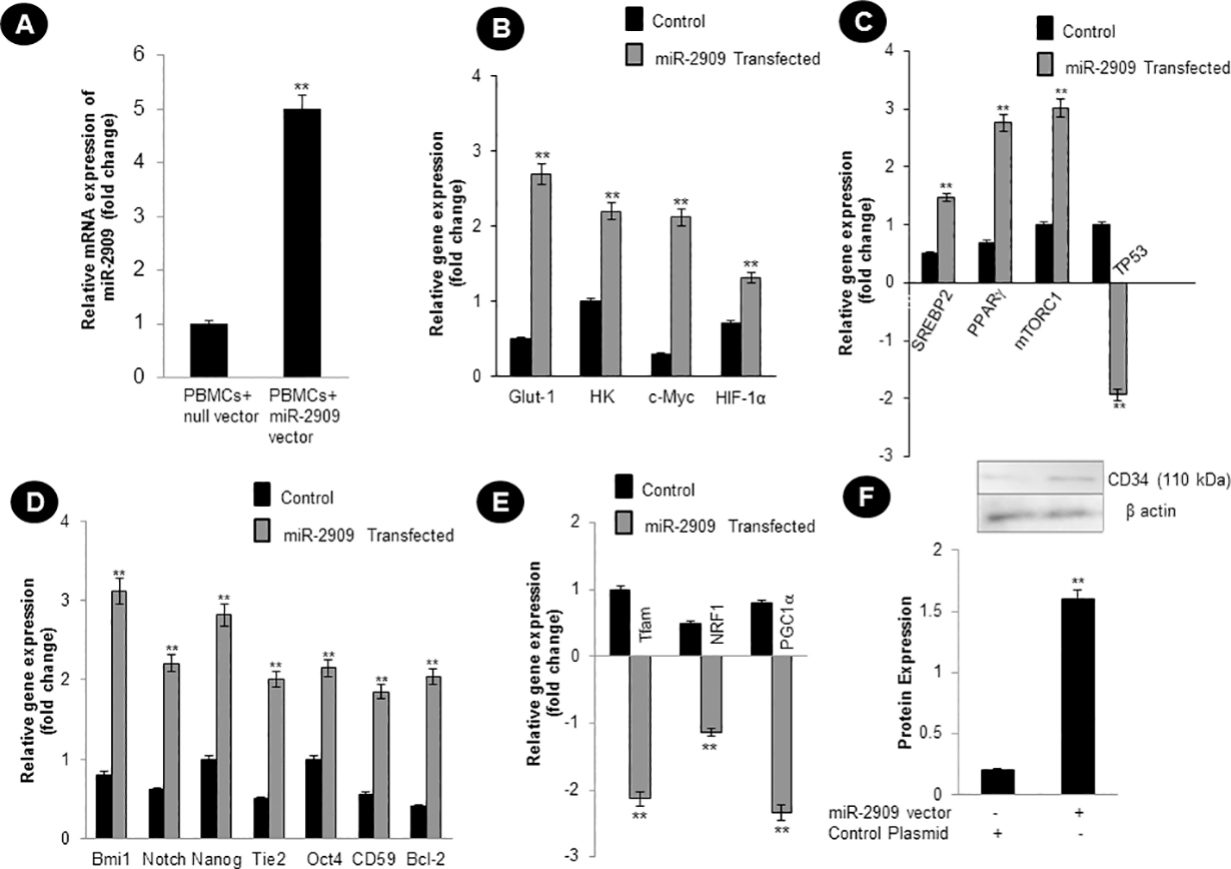
Expression of miR-2909 and its effector genes in human PBMCs. **(A)** Ectopic expression of miR-2909 within human PBMCs was measured in miR-2909 expression vector transfected cells as well as human PBMCs transfected with control vector containing scrambled sequence; U6 snRNA was used as an invariant control. **(B-E)** qRT-PCR analysis for relative expression of genes coding for *Glut-1*, *HK*, *c-Myc*, *HIF1α*, *SREBP2*, *PPARɣ*, *mTORC1*, *TP53*, *Bmi-1*, *Notch Tie-2*, *Oct-4*, *CD59*,*BCl-2*, *Tfam*, *NRF1*, *PGC1α* in human PBMCs exhibiting miR-2909 overexpression as well as in human PBMCs transfected with control vector containing scrambled sequence; GAPDH was used as an invariant control for studying the effector genes expression. **(F)** Translational expression of CD34 gene was studied in human PBMCs that were ectopically expressing miR-2909 as compared to corresponding control cells. Densitometry analysis of western blot was performed using Scion Image Analysis Software and the result was expressed as intensity ratio of CD34 protein to β-actin protein taken as AU (arbitrary unit. Each bar represents mean ± S.D of all the experiments performed in triplicate indicating 3 separate transfections, **p < 0.01 with respect to control.

In our previous study [3] we have shown that the ectopic expression of miR-2909 within human PBMCs results in the upregulation of mitochondrial uncoupling (*UCP2*) gene which favours aerobic glycolysis as the energy driving force for such cells. The present study revealed that the miR-2909 ectopic expression within human PBMCs (Fig 1A) results in the upregulation of genes (Fig 1B) coding for *c-Myc*, *HIF-1α*, glucose transporter-1 (*Glut-1*), hexokinase (*HK*) that have intrinsic ability to favour aerobic glycolysis. This phenomenon induced by miR-2909was paralleled by downregulation of *p53* (Fig 1C), widely known to favour aerobic respiration and restrict aerobic glycolysis [24,25]. Interestingly, miR-2909 RNomics was also found to upregulate genes coding for *mTORC1*, *SREBP2* and *PPARγ* (Fig 1C). It is pertinent to note here that *mTORC1* is known to upregulate genes coding for *c-Myc*, *HIF-1α*, *SREBP2* and *PPARγ* [6,7]. Hence from our results, it is clear that miR-2909 dependent-gene regulation ensures cellular aerobic glycolysis coupled with increased lipid synthesis through the expression of genes coding for *SREBP2 and PPARγ* (Fig 1C).Next we analysed the expression of transcriptional regulators of mitochondrial OXPHOS genes, decreased expression of *Tfam*, *NRF1*, *PGC1α* in miR-2909 expression vector transfected human PBMCs clearly exhibits that miR-2909 transfected PBMCs restricts aerobic respiration favouring aerobic glycolysis in contrast to human PBMCs transfected with control vector containing scrambled sequence displays favouring aerobic respiration and restricting aerobic glycolysis (Fig 1D). Further, since aerobic glycolysis within somatic cells ensures their dedifferentiation into stem-like phenotype [1] the present study was directed to explore if miR-2909 RNomics has the ability to up-regulate the genes known to play crucial role in the maintenance of stem-like phenotype. Such a study indeed revealed the ability of miR-2909 RNomics to upregulate genes coding for *Bmi-1*, *Notch*, *Nanog*, *Tie-2*, *Oct4*,*CD59*, *Bcl-2*and *CD34* (Fig 1E and 1F) within human PBMCs. The upregulation of these genes that define stem-like phenotype of cells as well as their survival attest to the fact that miR-2909 RNomics has the ability to induce dedifferentiation of somatic cells. This phenomenon is also attested by the fact that the miR-2909 RNomicsis also accompanied by the downregulation of *p53* gene (widely known as barrier for dedifferentiation (Fig 1C), [9,10].

### Ectopic Cellular miR-2909 expression ensures mitochondrial remodelling

**Fig 2:**
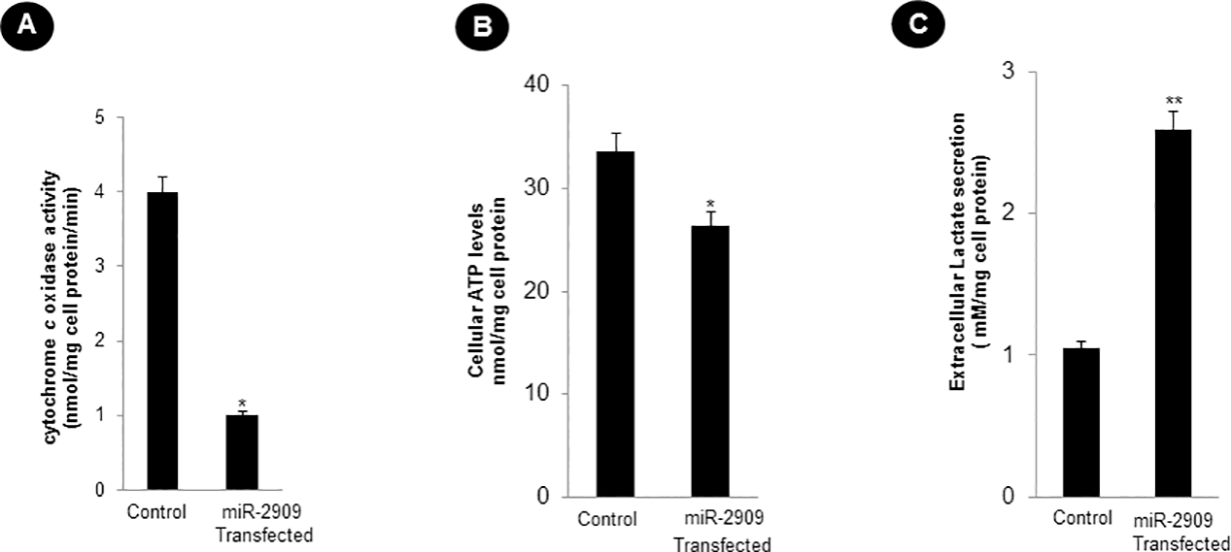
Cellular miR-2909 ectopic expression compromises mitochondrial function. **(A)** Mitochondrial cytochrome c oxidase activity, **(B)** cellular ATP levels and **(C)** extracellular secretion of lactate within human PBMCs transfected with miR-2909 expression vector compared with control null vector containing scrambled sequence using their respective kits. Each bar represents mean ± S.D of all the experiments performed in triplicate indicating 3 separate transfections, **p < 0.01, *p < 0.05 with respect to control. Bradford assay was used to quantify the protein concentration.

**Fig 3:**
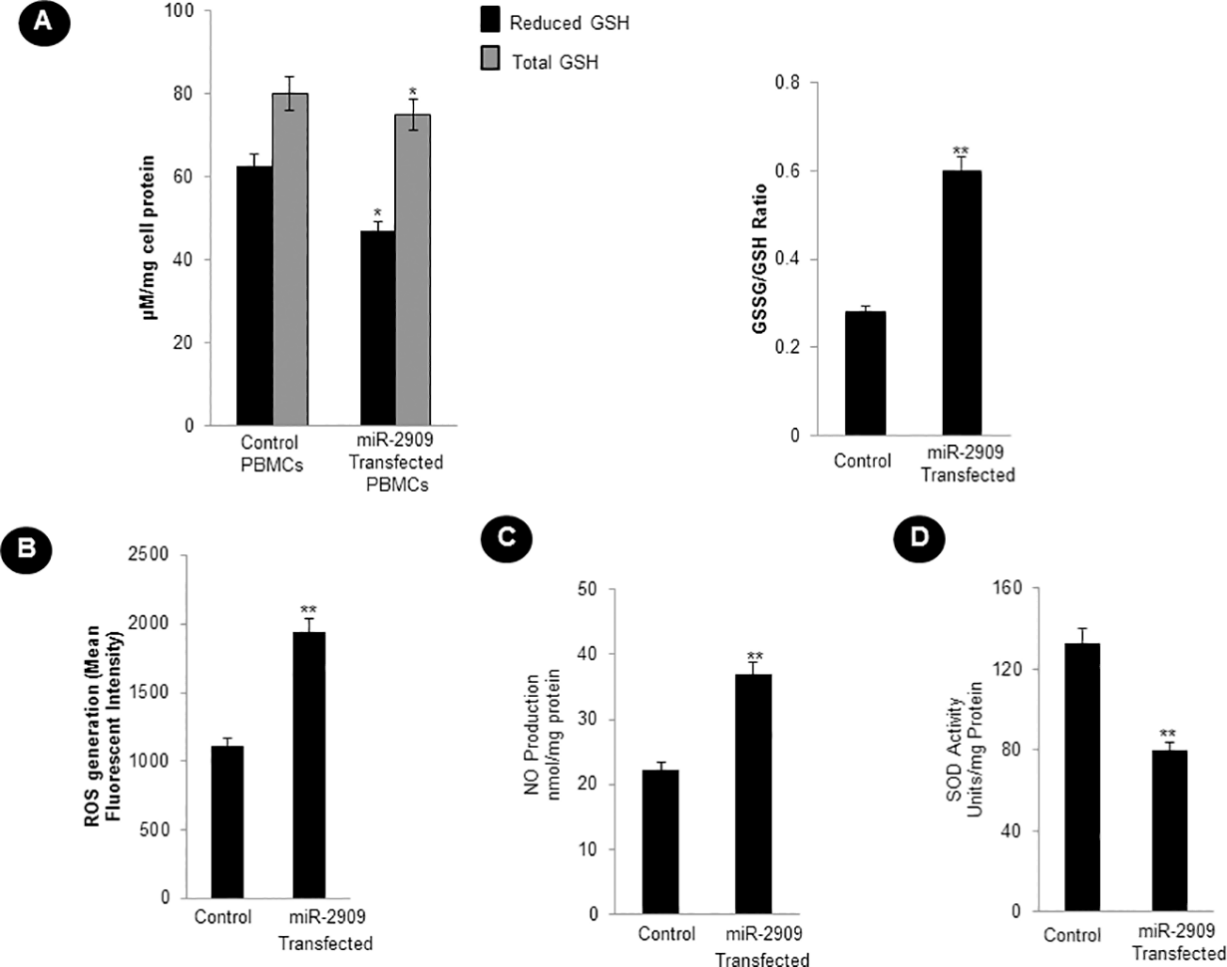
Cellular miR-2909 ectopic expression augments oxidative stress. **(A)** Glutathione oxidized (GSSG) to Glutathione reduced (GSH) ratio (GSSG to GSH) as a marker for oxidative stress, **(B)** flow-cytometric detection of reactive oxygen species (ROS) generation, **(C)** nitric oxide **(**NO) measurement and **(D)** superoxide dismutase(SOD) activity within human PBMCs transfected with miR-2909 expression vector compared with control null vector containing scrambled sequence after 48h incubation period. Each bar represents mean ± S.D of all the experiments performed in triplicate indicating 3 separate transfections, **p < 0.01, *p< 0.05 with respect to control.

Since the transition of somatic cells into stem-like phenotype is associated with mitochondrial remodelling, it became pertinent to examine the effect of cellular miR-2909 RNomics on ATP and lactate production as well as cytochrome *c* oxidase activity. Such a study revealed that ectopic miR-2909 expression within human PBMCs reduced their cytochrome *c* oxidase activity by about 4-fold (Figs 2A and S1A Fig) and ATP levels significantly by about 22% (Fig 2B).This phenomenon within such cells was accompanied by 2.5 fold increase in the lactate secretion (Fig 2C); two-fold increase in GSSG/GSH ratio, reactive oxygen species (ROS) coupled with nitric oxide (NO) production as well as decreased superoxide dismutase (SOD) activity (Fig 3 and S1B Fig). This study confirmed the ability of miR-2909 RNomics to induce altered mitochondrial function as a result of restricted aerobic respiration.

### Cellular ectopic miR-2909 expression ensures de-differentiation and stem-cell like state

**Fig 4:**
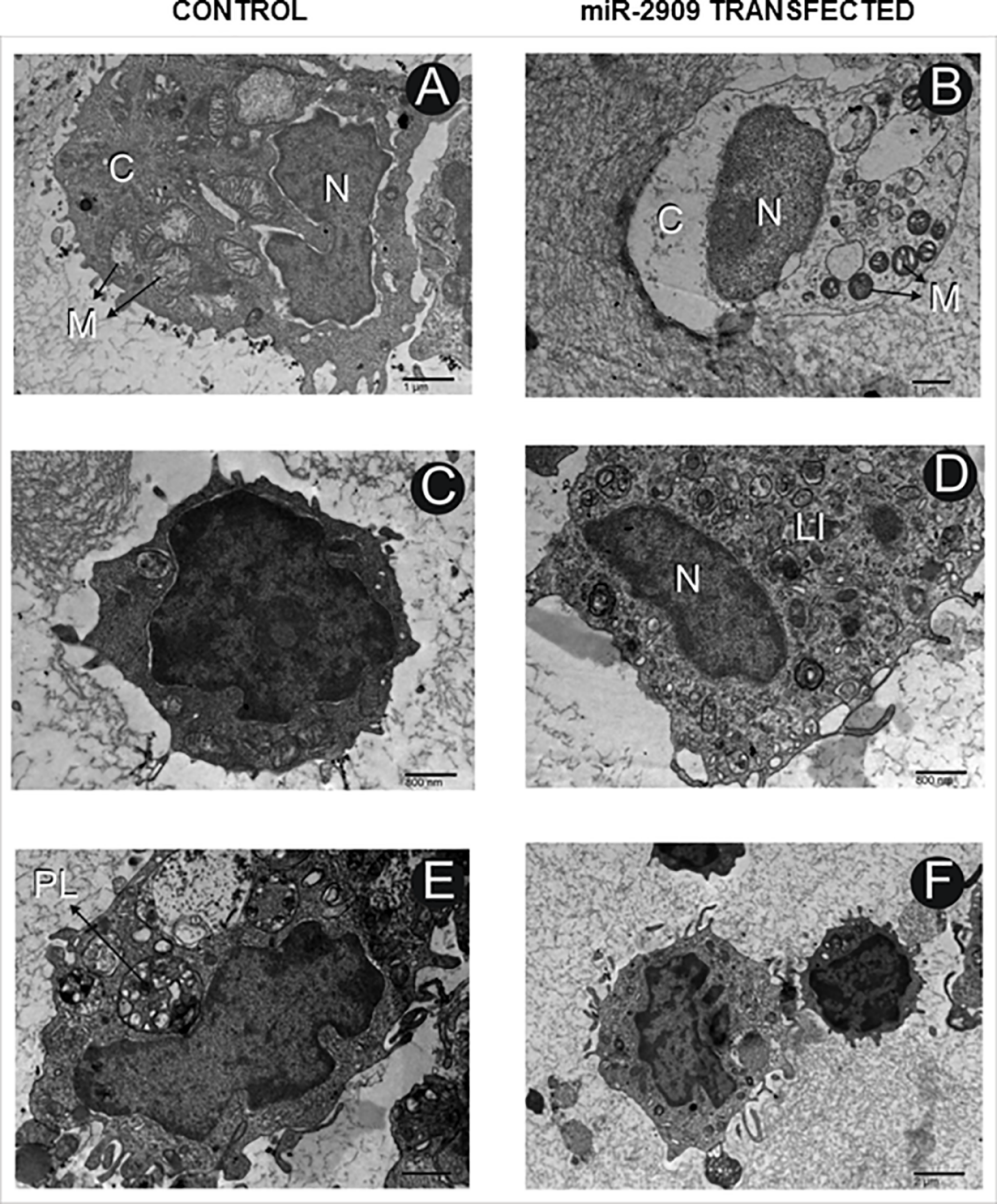
miR-2909 induced cellular ultrastructural traits. **(A-B)** Representative images of electron microscopy (TEM) of human PBMCs transfected with null vector containing scrambled sequence compared with miR-2909 expression vector. Globular shaped mitochondria (M) coupled with cristae-poor morphology in human PBMCs transfected with miR-2909 expression vector in contrast to normal elongated mitochondrial morphology with well aligned lamellar cristae in human PBMCs transfected with control null vector containing scrambled sequence. **(C-D)** Increased formation of intracellular lipid inclusion bodies (LI) in human PBMCs transfected with miR-2909 expression vector compared with control null vector containing scrambled sequence transfected human PBMCs. Scale 800nm;Final Magnification 14110X. **(E-F)** 50cells/case was studied under TEM for the observation of apoptotic changes in human PBMCs transfected with control vector and miR-2909 expression vector after 48h incubation period. We observed that few cells under TEM were indicating early apoptotic changes and formation of phagolysosome (PL) in human PBMCs transfected with control vector however no apoptotic changes were observed in healthy PBMCs transfected with miR-2909 expression vector. Stain: Uranyl acetate and lead citrate.

**Fig 5:**
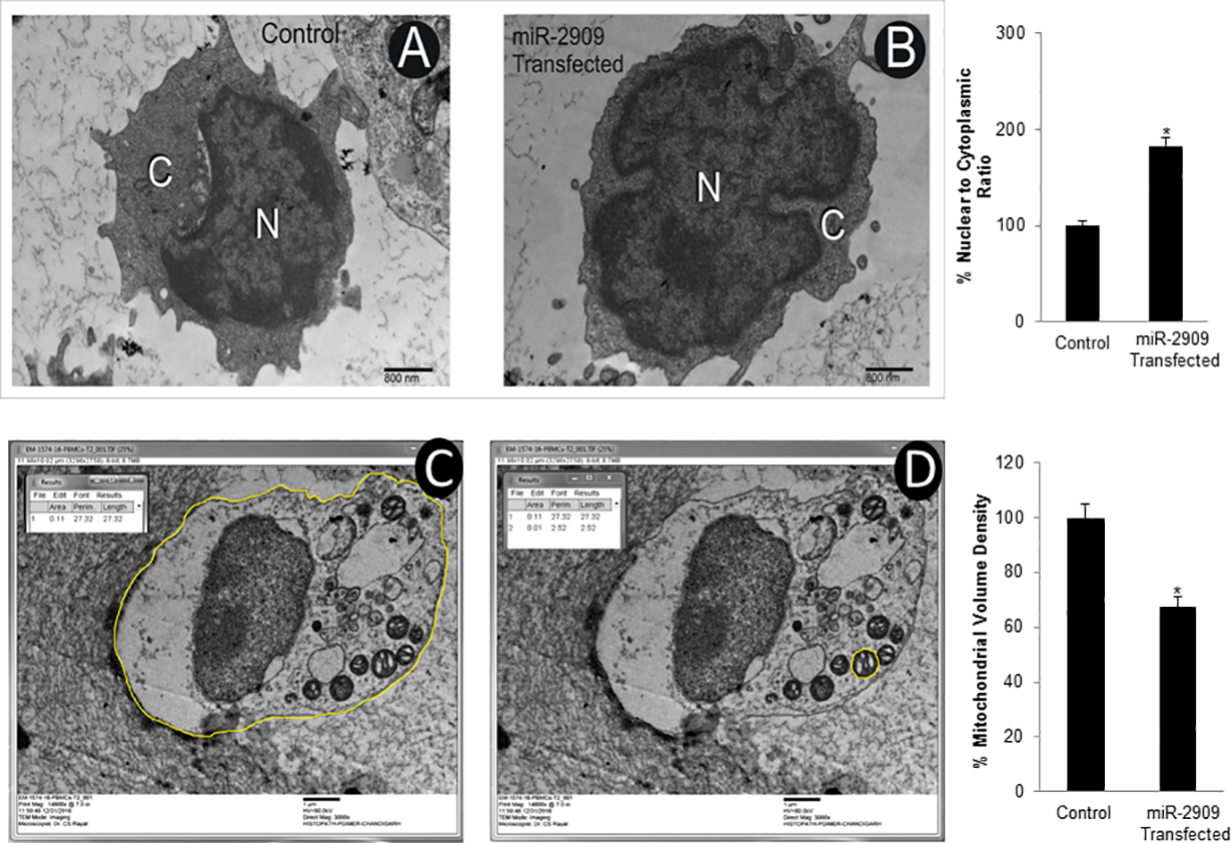
mIR-2909 influenced nuclear/cytoplasmic ratio and mitochondrial volume density as exhibited by morphometric analysis of an electron micrograph. **(A-C)** Mean nuclear/cytoplasmic (N/C) ratio (taken randomly 12 cells in both control PBMCs transfected with null vector containing scrambled sequence and miR-2909 transfected PBMCs) was calculated using Image J software equipped with XR81M-B camera after 48h incubation period. Scale 800nm, Final Magnification 14110X. **(D-E)** Representative ultrastructural features of a PBMC depicting measurement of an area of cell and mitochondrion (M) indicated by yellow line. Mean mitochondrial volume density of 12 cells (taken randomly 12 cells in both control PBMCs transfected with null vector containing scrambled sequence and miR-2909 transfected PBMCs) was expressed as percent volume: Vv = (Pm/Pc)*100 (%), where Pm is the pixel size occupied by each mitochondrial structure and Pc is the total cell mass in pixel size occupied by the cell calculated using Image J software equipped with XR81M-B camera. Error bars represent mean ± S.D. *p< 0.05with respect to control. Scale 1μm, Final Magnification 8460X, Staining: Uranyl acetate and lead citrate.

**Fig 6:**
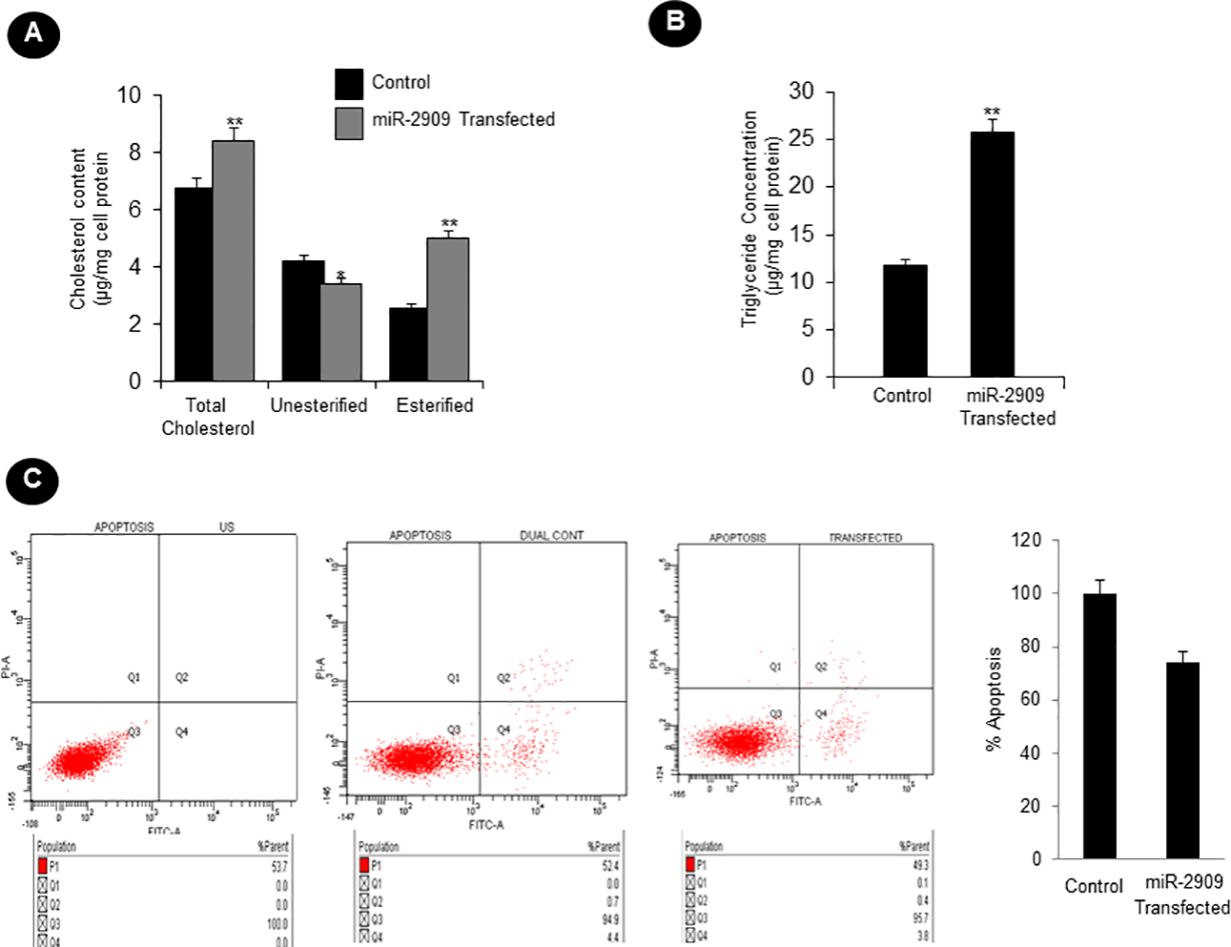
Influence of miR-2909 on lipogenesis and apoptosis. **(A-B)** Estimation of lipid inclusions rich in cholesterol esters and triglyceride concentration in human PBMCs transfected with either miR-2909 expression vector or control vector after 48h incubation period. **(C-D)** Flow-cytometric detection of apoptosis within human PBMCs transfected with either miR-2909 expression vector or control vector after 48h incubation period. US: Unstained, Dual Control: Human PBMCs transfected with null vector containing scrambled sequence, Transfected: Human PBMCs transfected with miR-2909 expression vector. Each bar represents mean ± S.D of all the experiments performed in triplicate indicating 3 separate transfections **p < 0.01, *p <0.05 relative to control.

Since reprogramming of somatic cells into their stem-like phenotype has been widely shown to be associated with mitochondrial remodelling, aerobic glycolysis and increased cellular nuclear to cytoplasmic (N/C) ratio as well as lipid inclusion bodies [1], the present study was also directed to examine the ultrastructural characteristics of human PBMCs having ability to ectopically express miR-2909. Such an ultrastructural study revealed that these miR-2909 programmed PBMCs contained mitochondria (M) exhibiting globular and cristae-poor morphology as well as intracellular lipid-inclusion bodies (LI) in contrast to corresponding control PBMCs with normal ultrastructural features (Fig 4A–4D). Mean nuclear to cytoplasm (N/C) ratio and mean mitochondrial volume density was calculated in 12 cells (taken randomly from both miR-2909 transfected human PBMCs and control PBMCs transfected with scrambled sequence) using Image J software and results showed that cellular N/C ratio increased by approximately two fold and mitochondrial volume density decreased by 26.09% in miR-2909 transfected PBMCs compared with control PBMCs (Fig 5 and S3 Fig). Lipid-inclusion bodies (LI) rich in triglycerides and esterified-cholesterol were estimated and high content was observed inmiR-2909 transfected PBMCs compared with control PBMCs (Fig 6A and 6B). Hence miR-2909 RNomics has the inherent capacity to reprogram human PBMCs into stem-like phenotype. This view is further strengthened by the observation miR-2909 induced altered mitochondrial function as well as upregulation of genes that play crucial role in promoting aerobic glycolysis, dedifferentiation, lipid synthesis and stem-cell like state within human PBMCs. We studied 50 cells/case under TEM for the observation of apoptotic changes in human PBMCs transfected with control vector and human PBMCs transfected with miR-2909 expression vector after 48h incubation period. We observed that few cells under TEM were indicating early apoptotic changes and formation of phagolysosome (PL) in human PBMCs transfected with control vector however no apoptotic changes were observed in healthy PBMCs transfected with miR-2909 expression vector (Fig 4E and 4F and S4 Fig). This finding was further strengthened via FACS analysis when ectopic expression of miR-2909 within human PBMCs exhibited non-significant reduction in apoptosis compared with control PBMCs transfected with null vector containing scrambled sequence (Fig 6C and 6D).

## Discussion

Like bacteria, the human cells are capable of reverting to a more primitive form as and when their aerobic respiration is curtailed and switched to aerobic glycolysis in order to produce metabolites of glycolysis for growth and survival [26,27]. While recent advances have started to provide insights into these energy metabolic states, we have little or no understanding of the driving mechanism that is responsible for the transition of functionally mature mitochondria into its immature state and vice-versa during reprogramming and differentiation respectively [1]. In this context, the regulation of two genes coding for *p53* and mitochondrial uncoupling protein (*UCP2*) assumes importance because *UCP2* initiates aerobic glycolysis by shunting pyruvate out of mitochondria [1–5] and upregulation of *p53* expression restricts aerobic glycolysis and favours aerobic respiration [24,25]. It is pertinent to take note of AATF gene which holds AATF protein coding transcript and regulatory non-coding miR-2909 within its fold to exhibit circadian rhythmicity as a result of mutually cooperative regulation [28]. AATF protein ensures sustained expression of miR-2909 through the activation of NFκB and induces *p53* gene expression as well as influences *p53* protein stability through the induction of genes coding for *c-Myc and Bmi-1* [12, 29,30]. The miR-2909 on the other hand ensures sustained expression of genes coding for *AATF*, *c-Myc*, *UCP2* and *Bmi-1* as well as downregulation of *p53* gene [12,29,30]. This bi-stable cooperative expression of *AATF* gene and its encoded miR-2909 ensures regulation of *UCP2* in a cyclic fashion [12] thereby allowing human cells to decouple/couple the glycolysis from/with the aerobic respiration in a fashion that equip these cells with the ability to convert functionally mature mitochondria into an immature state and vice-versa during reprogramming of somatic cells into stem-like phenotype and differentiation respectively. It is in this context, the results reported here assume importance because ectopic expression of miR-2909 within human PBMCs resulted in their reprogramming into stem-like phenotype indicated by mitochondrial globular shaped coupled with cristae-poor morphology, increased nuclear to cytoplasmic ratio, increased lactate production, low ATP levels, significant reduction in mitochondrial cytochrome *c* oxidase (COX) activity recognized widely as the rate-limiting step in cellular aerobic-respiration [31,32], increased production of cellular oxidative stress (Figs 1–6). This miR-2909 induced cellular phenomenon was paralleled by the downregulation of p53 gene (known widely to restrict somatic cell reprogramming into stem-like phenotype; [9,10]) as well upregulation of genes that play crucial role in cellular dedifferentiation and survival (Fig 1C, 1E and 1F). The ability of miR-2909 RNomics to upregulate *mTORC1* gene ensures enhanced cellular lipogenesis (via its positive regulation of *SREBP2*) as well as increased formation of intracellular lipid droplets rich in esterified cholesterol and triglycerides via positive regulation of *PPARγ* gene (Figs 1C, 6A and 6B). This phenomenon of *mTORC1* induced cellular lipid biosynthesis and storage is further strengthened by the fact that *mTORC1* has been shown to inhibit lipophagy mainly through the inhibition of autophagy and lysosome biogenesis [33,34]. All these above-mentioned findings taken together attest to the ability of miR-2909 RNomics to reprogram the human PBMCs into stem-like phenotype by ensuring mitochondrial remodelling and aerobic glycolysis coupled with intracellular lipid inclusion bodies. This conclusion is further strengthened by the findings that revealed the influence of miR-2909 upon the mitochondrial OXPHOS genes as well as on the cellular production and secretion of lactate (Figs 1D and 2C).

## Supporting information

S1 Table List of Primer sequences used in the study. (DOCX)

S1 Fig Cellular miR-2909 ectopic expression compromises mitochondrial cytochrome c oxidase activity and augments ROS generation. **(A)** Decrease in OD at 550nm was observed in human PBMCs transfected with null vector containing scrambled sequence in contrast to no change in OD in human PBMCs transfected with miR-2909 expression vector. The decrease in OD was recorded over a period of 45 min using automatic plate reader. The rate of the reaction was calculated in the linear range by subtracting the initial OD reading from the final OD, t1 and t2 represents linear rate of reaction. **(B)** Flow-cytometric detection of reactive oxygen species (ROS) generation in human PBMCs transfected with null vector containing scrambled sequence compared with corresponding control cells transfected with miR-2909 expression vector.
(DOCX)

S2 Fig Relative expression of miR-2909 in human PBMCs following different transfections. qRT-PCR analysis for relative expression of miR-2909 in human PBMCs transfected with null vector containing scrambled sequence, human PBMCs transfected with miR-2909 expression vector and human PBMCs co-transfected with miR-2909 expression vector and antagomiR-2909 expression vector.
(DOCX)

S3 Fig Effect of miR-2909 knock-down on nuclear/cytoplasmic ratio. **(A)** Representative TEM images indicating N/C ratio in human PBMCs transfected with null vector containing scrambled sequence, **(B)** human PBMCs transfected with miR-2909 expression vector and **(C)** human PBMCs co-transfected with miR-2909 expression vector and antagomiR-2909 expression vector. N/C ratio was calculated using Image J software.
(DOCX)

S4 Fig Effect of miR-2909 knock-down on cellular ultrastructural features of human PBMCs. Representative images of electron microscopy (TEM) of human PBMCs transfected with null vector containing scrambled sequence, human PBMCs transfected with miR-2909 expression vector and human PBMCs co-transfected with miR-2909 expression vector and antagomiR-2909 expression vector. **(A-B)** Globular shaped mitochondria (M) coupled with cristae-poor morphology in human PBMCs transfected with miR-2909 expression vector in contrast to normal elongated mitochondrial morphology with well aligned lamellar cristae in human PBMCs transfected with control null vector containing scrambled sequence. **(C)** However no appreciable change in mitochondrial morphology was observed in healthy PBMCs co-transfected with miR-2909 expression vector and antagomiR-2909 vector. **(D-E)** Increased formation of intracellular lipid inclusion bodies (LI) and myelin figures (MF) in human PBMCs transfected with miR-2909 expression vector compared with human PBMCs transfected with control null vector containing scrambled sequence. **(F)** However on co-transfection of human PBMCs with E2F-miR-2909 expression vector and antagomiR-2909 vector around 60% of cells showed depletion of intracellular lipid inclusion bodies. Scale 800nm; Final Magnification 14110X. 50cells/case was studied under TEM for the observation of apoptotic changes in human PBMCs transfected with above-mentioned expression vectors after 48h incubation period. (**G,I**) We observed that few cells under TEM were indicating early apoptotic changes and formation of phagolysosome (PL) in human PBMCs transfected with control null vector containing scrambled sequence and human PBMCs co-transfected withmiR-2909 expression vector and antagomiR-2909 expression vector **(H)** however no apoptotic changes were observed in healthy PBMCs transfected with miR-2909 expression vector.
(DOCX)
