# Supplementary material for: Human cellular mitochondrial remodelling is governed by miR-2909 RNomics
Source: PLoS One. 2018 Sep 25;13(9):e0203614. doi: 10.1371/journal.pone.0203614 (PMC6155498; doi:10.1371/journal.pone.0203614)
Supplement: S1 Table — (DOCX) [file pone.0203614.s001.docx]

| **Gene** | **Primer sequence (5’-3’)** |
| --- | --- |
| miR-2909 | F: TTTTACTTGAATCCCTTGAGGTG  R: AGCTGGAGAACAATGCTGGT |
| U6 | F: CGCTTCGGCAGCACATATACTAA  R: TATGGAACGCTTCACGAATTTGC |
| GAPDH | F: CATGTACGTTGCTATCCAGGC  R: CTCCTTAATGTCACGCACGAT |
| GLUT-1 | F: TGAGCATCGTGGCCATCTTT  R: CCGGAAGCGATCTCATCGAA |
| HK-2 | F: AGAGAGGACCCCACTGGACT  R: CCAAGGTGAAGCAACCGTAT |
| HIF-1α | F: CCCAATGGATGATGACTTCC  R: TGGGTAGGAGATGGAGATGC |
| c-Myc | F: CCAGCAGCGACTATGAGG  R: CCAAGACGTTGTGTGTTC |
| P53 | F: GAAGACCCAGGTCCAGATGA  R: CTGCCCTGGTAGGTTTTCTG |
| mTORC1 | F: CCAACAGTTCACCCTCAGGT  F: GCTGCCACTCTCCAAGTTTC |
| SREBP2 | F: CAGCAGTCTCTGAGCACCAG  R: CCCTGGCTGTCCTGTGTAAT |
| Bmi-1 | F: TCTGCAGCTCGCTTCAAGAT  R: AGTGGTCTGGTCTTGTGAAC |
| CD59 | F: GGGGCAGGAGTATATGAGCA  R: TCCCTGCCAGAAGTCCTCTA |
| Nanog | F: GTCTTCTGCTGAGATGCCTCACA  R: CTTCTGCGTCACACCATTGCTAT |
| NRF1 | F: CTTACAAGGTGGGGGACAGA  R:CAATGTCACCACCTCCACAG |
| Tfam | F: GTGGGAGCTTCTCACTCTGG  R: TAGGGCTTTTTCTCCTGCAA |
| PGC1α | F: AGCTGCTGAAGAGGCAAGAG  R: TTCCCCTAAACCAAGCACAC |

**S1 Table**. **List of Primer sequences used in the study.**
