# Supplementary material for: Human cellular mitochondrial remodelling is governed by miR-2909 RNomics
Source: PLoS One. 2018 Sep 25;13(9):e0203614. doi: 10.1371/journal.pone.0203614 (PMC6155498; doi:10.1371/journal.pone.0203614)
Supplement: S1 Fig — (A) Decrease in OD at 550nm was observed in human PBMCs transfected with null vector containing scrambled sequence in contrast to no change in OD in human PBMCs transfected with miR-2909 expression vector. The decrease in OD was recorded over a period of 45 min using automatic plate reader. The rate of the reaction was calculated in the linear range by subtracting the initial OD reading from the final OD, t1 and t2 represents linear rate of reaction. (B) Flow-cytometric detection of reactive oxygen species (ROS) generation in human PBMCs transfected with null vector containing scrambled sequence compared with corresponding control cells transfected with miR-2909 expression vector. (DOCX) [file pone.0203614.s002.docx]

**
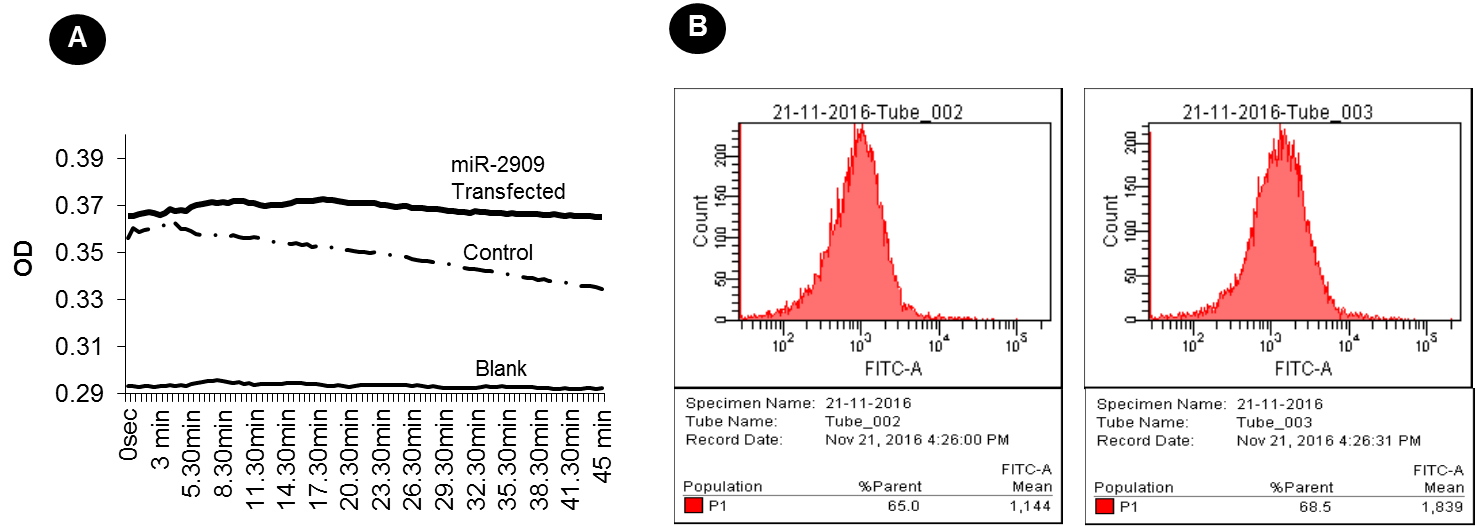
**

**S1 Fig. Cellular miR-2909 ectopic expression compromises mitochondrial cytochrome c** **oxidase activity and augments ROS generation**
