# Supplementary figures and images for: Human cellular mitochondrial remodelling is governed by miR-2909 RNomics
Source: PLoS One. 2018 Sep 25;13(9):e0203614. doi: 10.1371/journal.pone.0203614 (PMC6155498; doi:10.1371/journal.pone.0203614)

**
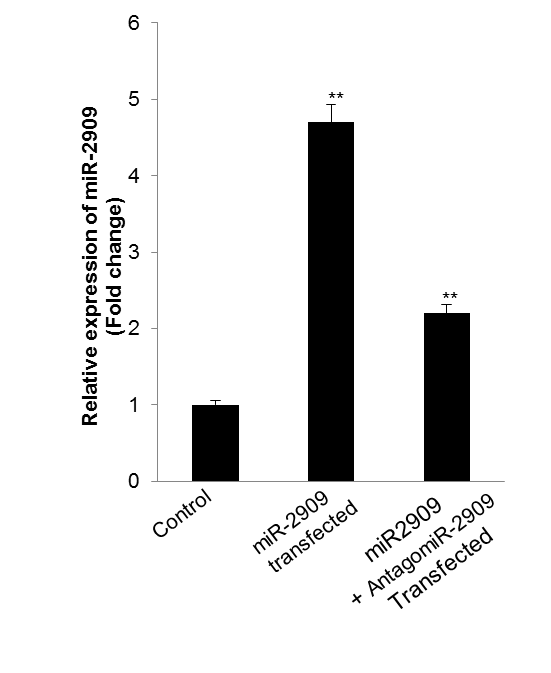
**

**S2 Fig** **Relative expression of miR-2909 in human PBMCs following different transfections**

Supplement: S2 Fig — qRT-PCR analysis for relative expression of miR-2909 in human PBMCs transfected with null vector containing scrambled sequence, human PBMCs transfected with miR-2909 expression vector and human PBMCs co-transfected with miR-2909 expression vector and antagomiR-2909 expression vector. (DOCX) [file pone.0203614.s003.docx]

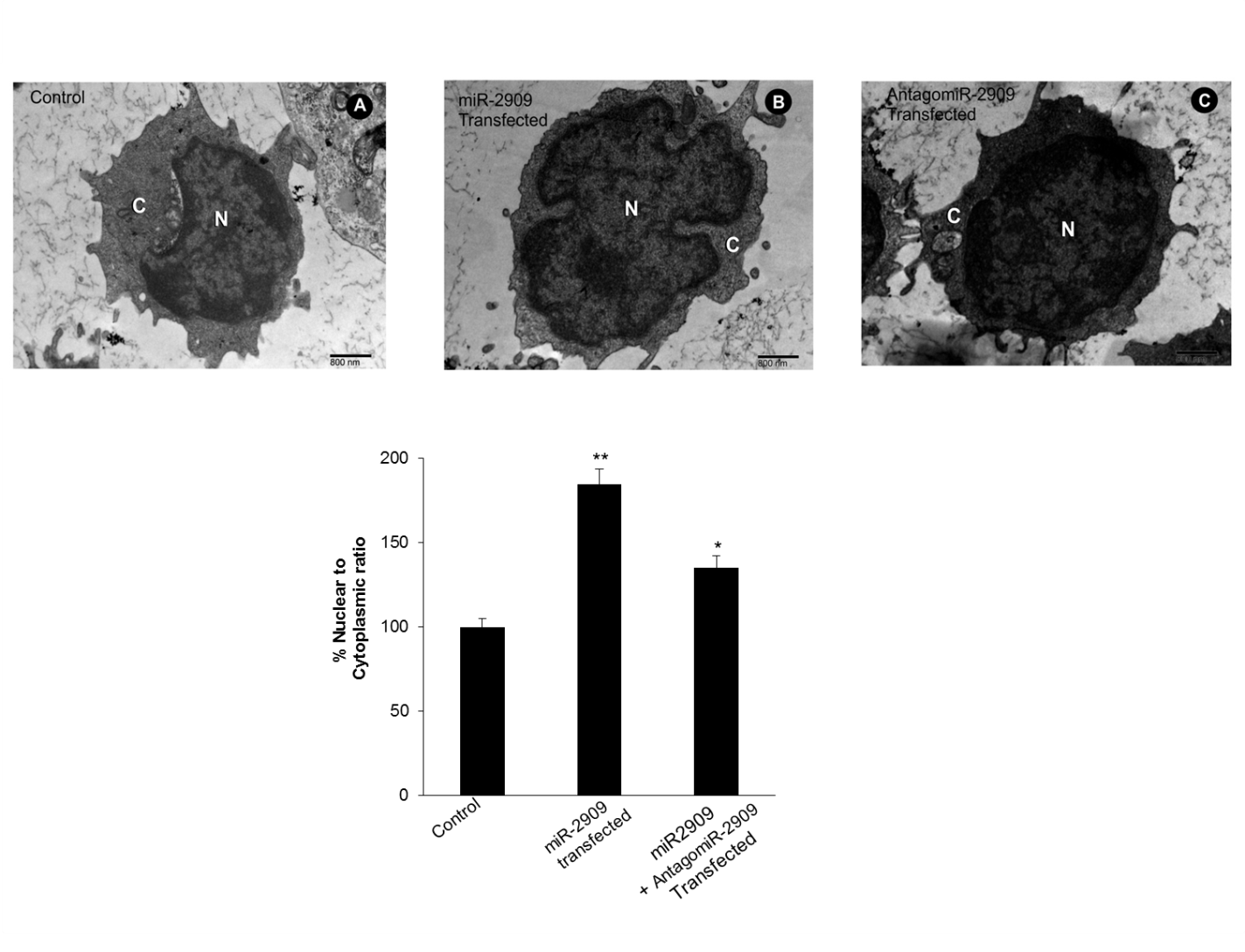


**S3 Fig. Effect of miR-2909 knock-down on nuclear/cytoplasmic ratio**

Supplement: S3 Fig — (A) Representative TEM images indicating N/C ratio in human PBMCs transfected with null vector containing scrambled sequence, (B) human PBMCs transfected with miR-2909 expression vector and (C) human PBMCs co-transfected with miR-2909 expression vector and antagomiR-2909 expression vector. N/C ratio was calculated using Image J software. (DOCX) [file pone.0203614.s004.docx]
