# Supplementary material for: Human cellular mitochondrial remodelling is governed by miR-2909 RNomics
Source: PLoS One. 2018 Sep 25;13(9):e0203614. doi: 10.1371/journal.pone.0203614 (PMC6155498; doi:10.1371/journal.pone.0203614)
Supplement: S4 Fig — Representative images of electron microscopy (TEM) of human PBMCs transfected with null vector containing scrambled sequence, human PBMCs transfected with miR-2909 expression vector and human PBMCs co-transfected with miR-2909 expression vector and antagomiR-2909 expression vector. (A-B) Globular shaped mitochondria (M) coupled with cristae-poor morphology in human PBMCs transfected with miR-2909 expression vector in contrast to normal elongated mitochondrial morphology with well aligned lamellar cristae in human PBMCs transfected with control null vector containing scrambled sequence. (C) However no appreciable change in mitochondrial morphology was observed in healthy PBMCs co-transfected with miR-2909 expression vector and antagomiR-2909 vector. (D-E) Increased formation of intracellular lipid inclusion bodies (LI) and myelin figures (MF) in human PBMCs transfected with miR-2909 expression vector compared with human PBMCs transfected with control null vector containing scrambled sequence. (F) However on co-transfection of human PBMCs with E2F-miR-2909 expression vector and antagomiR-2909 vector around 60% of cells showed depletion of intracellular lipid inclusion bodies. Scale 800nm; Final Magnification 14110X. 50cells/case was studied under TEM for the observation of apoptotic changes in human PBMCs transfected with above-mentioned expression vectors after 48h incubation period. (G,I) We observed that few cells under TEM were indicating early apoptotic changes and formation of phagolysosome (PL) in human PBMCs transfected with control null vector containing scrambled sequence and human PBMCs co-transfected withmiR-2909 expression vector and antagomiR-2909 expression vector (H) however no apoptotic changes were observed in healthy PBMCs transfected with miR-2909 expression vector. (DOCX) [file pone.0203614.s005.docx]

**CONTROL miR-2909TRANSFECTED miR-2909+AntagomiR-2909TRANSFECTED**
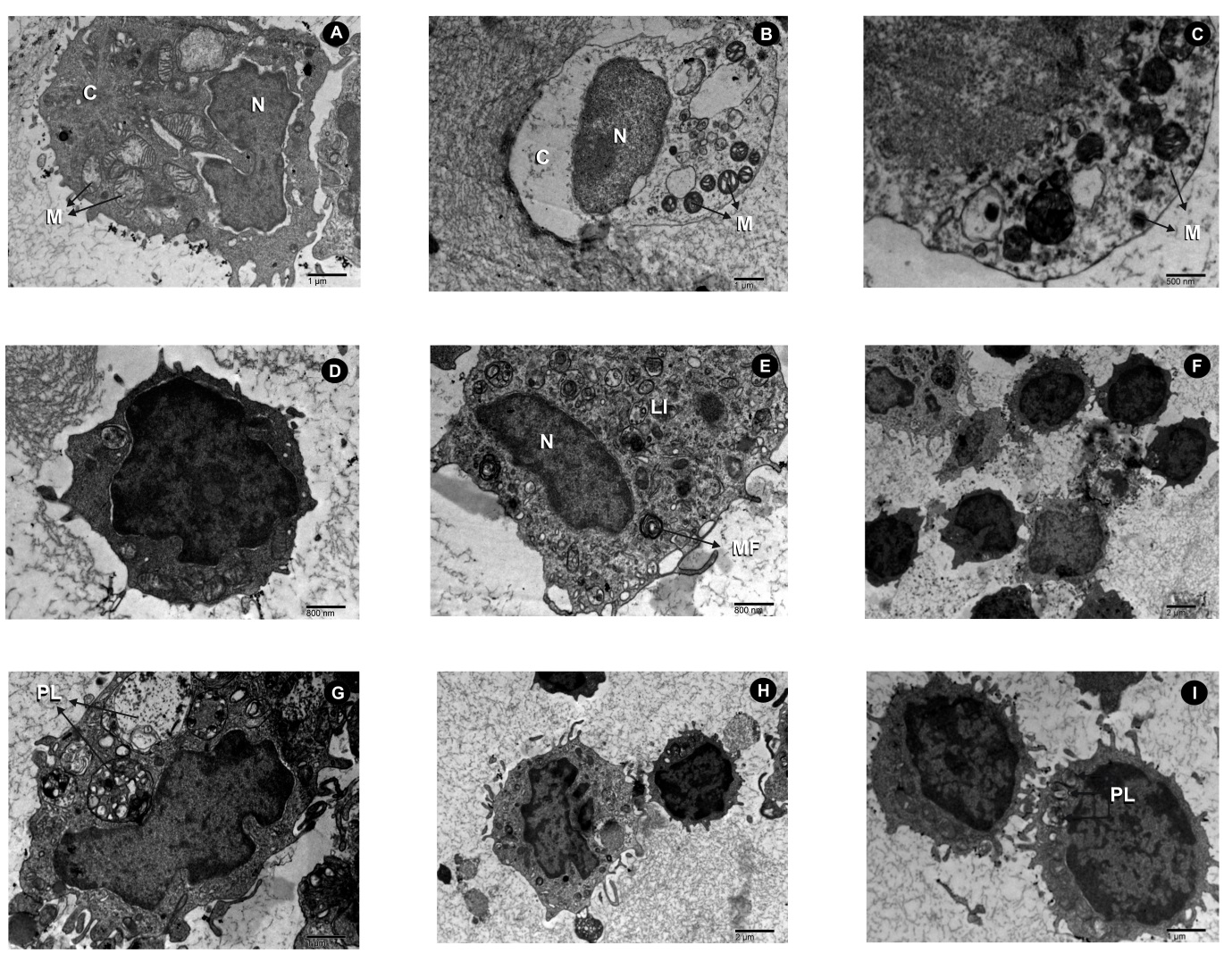


**S4 Fig. Effect of miR-2909 knock-down on cellular ultrastructural features of human PBMCs**
